# Assessment of the Potential Role of Muscle Spindle Mechanoreceptor Afferents in Chronic Muscle Pain in the Rat Masseter Muscle

**DOI:** 10.1371/journal.pone.0011131

**Published:** 2010-06-15

**Authors:** James P. Lund, Somayeh Sadeghi, Tuija Athanassiadis, Nadia Caram Salas, François Auclair, Benoît Thivierge, Isabel Arsenault, Pierre Rompré, Karl-Gunnar Westberg, Arlette Kolta

**Affiliations:** 1 Groupe de Recherche sur le Système Nerveux Central du Fonds de Recherche en Santé du Québec, Department of Physiology, Université de Montréal, Montréal, Québec, Canada; 2 Department of Stomatology, Université de Montréal, Montréal, Québec, Canada; 3 Faculty of Dentistry, McGill University, Montréal, Québec, Canada; 4 Section for Physiology, Department of Integrative Medical Biology, Umeå University, Umeå, Sweden; Emory University, United States of America

## Abstract

**Background:**

The phenotype of large diameter sensory afferent neurons changes in several models of neuropathic pain. We asked if similar changes also occur in “functional” pain syndromes.

**Methodology/Principal Findings:**

Acidic saline (AS, pH 4.0) injections into the masseter muscle were used to induce persistent myalgia. Controls received saline at pH 7.2. Nocifensive responses of Experimental rats to applications of Von Frey Filaments to the masseters were above control levels 1–38 days post-injection. This effect was bilateral. Expression of c-Fos in the Trigeminal Mesencephalic Nucleus (NVmes), which contains the somata of masseter muscle spindle afferents (MSA), was above baseline levels 1 and 4 days after AS. The resting membrane potentials of neurons exposed to AS (n = 167) were hyperpolarized when compared to their control counterparts (n = 141), as were their thresholds for firing, high frequency membrane oscillations (HFMO), bursting, inward and outward rectification. The amplitude of HFMO was increased and spontaneous ectopic firing occurred in 10% of acid-exposed neurons, but never in Controls. These changes appeared within the same time frame as the observed nocifensive behaviour. Ectopic action potentials can travel centrally, but also antidromically to the peripheral terminals of MSA where they could cause neurotransmitter release and activation of adjacent fibre terminals. Using immunohistochemistry, we confirmed that annulospiral endings of masseter MSA express the glutamate vesicular transporter VGLUT1, indicating that they can release glutamate. Many capsules also contained fine fibers that were labelled by markers associated with nociceptors (calcitonin gene-related peptide, Substance P, P2X3 receptors and TRPV1 receptors) and that expressed the metabotropic glutamate receptor, mGluR5. Antagonists of glutamatergic receptors given together with the 2^nd^ injection of AS prevented the hypersensitivity observed bilaterally but were ineffective if given contralaterally.

**Conclusions/Significance:**

Low pH leads to changes in several electrical properties of MSA, including initiation of ectopic action potentials which could propagate centrally but could also invade the peripheral endings causing glutamate release and activation of nearby nociceptors within the spindle capsule. This peripheral drive could contribute both to the transition to, and maintenance of, persistent muscle pain as seen in some “functional” pain syndromes.

## Introduction

Changes in the phenotype of large diameter spinal and trigeminal sensory afferents have been linked to the development of neuropathic pain [1]–[7], but little is known about the role of these afferents in the aetiology of the so-called “functional” pain syndromes [8] such as Fibromyalgia, Myofascial Pain and Temporomandibular Disorders (TMD). Interestingly, Weerakkody et al. [9] found in human volunteers, that vibration at a frequency optimal for stimulating muscle spindle group I afferents (80 Hz) decreases pressure-induced pain in the unexercised gastrocnemius muscle. However, in the presence of Delayed Onset Muscle Soreness (DOMS), induced by strenuous exercise several hours before, a similar vibration increased the intensity of pain induced by a corresponding pressure. Following several controls, it was concluded that the increased pressure-pain was due mainly to stimulation of the Ia afferents. In particular, it was found that a large fiber block applied by pressure to the sciatic nerve, that was just sufficient to eliminate the electrically evoked H-reflex, significantly increased the pressure-pain threshold during DOMS in the gastrocnemius. A similar observation had been previously reported for elbow flexor muscles by Barlas et al. [10]. DOMS symptoms, which have been considered to result from local damage and inflammation, usually resolve within one week when myofibrillar damage has healed. However, muscle injury has also been linked to longer lasting “functional” pain syndromes [11].

In order to mimic the pain associated with tissue acidosis and DOMS in humans, Issberner et al. [12] used single injections of acidic saline (pH 5.2) into forearm muscle tissue. Sluka et al. [13], [14] modified this method for use in rodents and showed that two injections of acidic saline into the gastrocnemius muscle of rats 2 to 5 days apart produce mechanical hyperalgesia (hereafter only referred to as “allodynia” or “allodynia-like” responses) that lasts for several weeks. To further investigate the possible contribution of muscle spindle afferents to DOMS and to persistent muscle pain states, we chose to use the masseter muscle as our model. The large-diameter (Ia) muscle spindle afferents of this jaw closing muscle can be positively identified by retrograde labelling, because their cell bodies are located within the Trigeminal Mesencephalic Nucleus (NVmes) [15], [16]. All the other groups of masseter afferents have their cells bodies in the Trigeminal Ganglion (TG). Morphologically, the NVmes neurons resemble large dorsal root ganglia (DRG) cells, and they have similar specific intrinsic electrical properties: inward rectification on membrane hyperpolarization [17]–[19] and high frequency subthreshold membrane oscillations [19]–[21]. An increase in the amplitude of these oscillations leads to repetitive firing in NVmes cells [19]. Previous studies on neuropathic pain have revealed that increases in oscillation amplitude of dorsal root ganglion neurons (DRG) and spontaneous ectopic firing in cutaneous and muscle afferent fibers contribute to both peripheral and central sensitization of nociceptive pathways [22].

Here we tested the hypothesis that similar changes may also occur under less severe pain conditions, like those induced in the acidic saline model, and hypothesized that action potentials that could be generated at the soma by an increase in oscillation amplitude could travel antidromically to the peripheral terminals of the axon and cause release of glutamate. If nociceptive fibres carrying glutamate receptors lie nearby, they could be activated by the released glutamate. It was shown recently that annulospiral endings of muscle spindles of the masseter [23] and other muscles [24] express the vesicular glutamate transporter VGLUT1, suggesting that they can release glutamate. The existence of small-diameter axons within muscle spindles and other muscle mechanoreceptors including tendon organs, Ruffini endings and Meissner corpuscles has been known for many decades, but has been assumed to represent sympathethic innervation [25]–[27]. In the case of muscle spindles, Matthews [25] wrote that “the main afferent may be accompanied by a finer accessory nerve fibre… whether to supply intracapsular blood vessels or to mediate “pain” is quite uncertain”.

In our series of studies, we first used behavioural testing to validate the use of acidic saline to induce long lasting allodynia in masseter muscles. Investigation of the electrophysiological properties of NVmes cells exposed to acidic saline revealed changes in their oscillations and membrane properties and increased spontaneous ectopic firing during the same time frame as the qobserved behavioural allodynia. The acidic saline muscle injection also increased the expression of the early gene c-Fos in NVmes neurons, which further indicates that long term changes in activation of these neurons appear after the muscle injections. Using immunofluorescence , we confirmed that annulospiral endings of masseter spindle Ia afferents have the capacity to release peripherally-stored glutamate, which could activate adjacent nociceptors that express glutamate receptors. We also showed that blockade of these peripheral glutamate receptors prevents development of the acid-induced allodynia. Together our observations support a hypothesis that activity in inflicted Ia afferents should be considered a factor that may favor the development and maintenance of persistent oro-facial muscle pain.

## Results

### Acidic saline injections increase nocifensive behaviour

**Figure 1:**
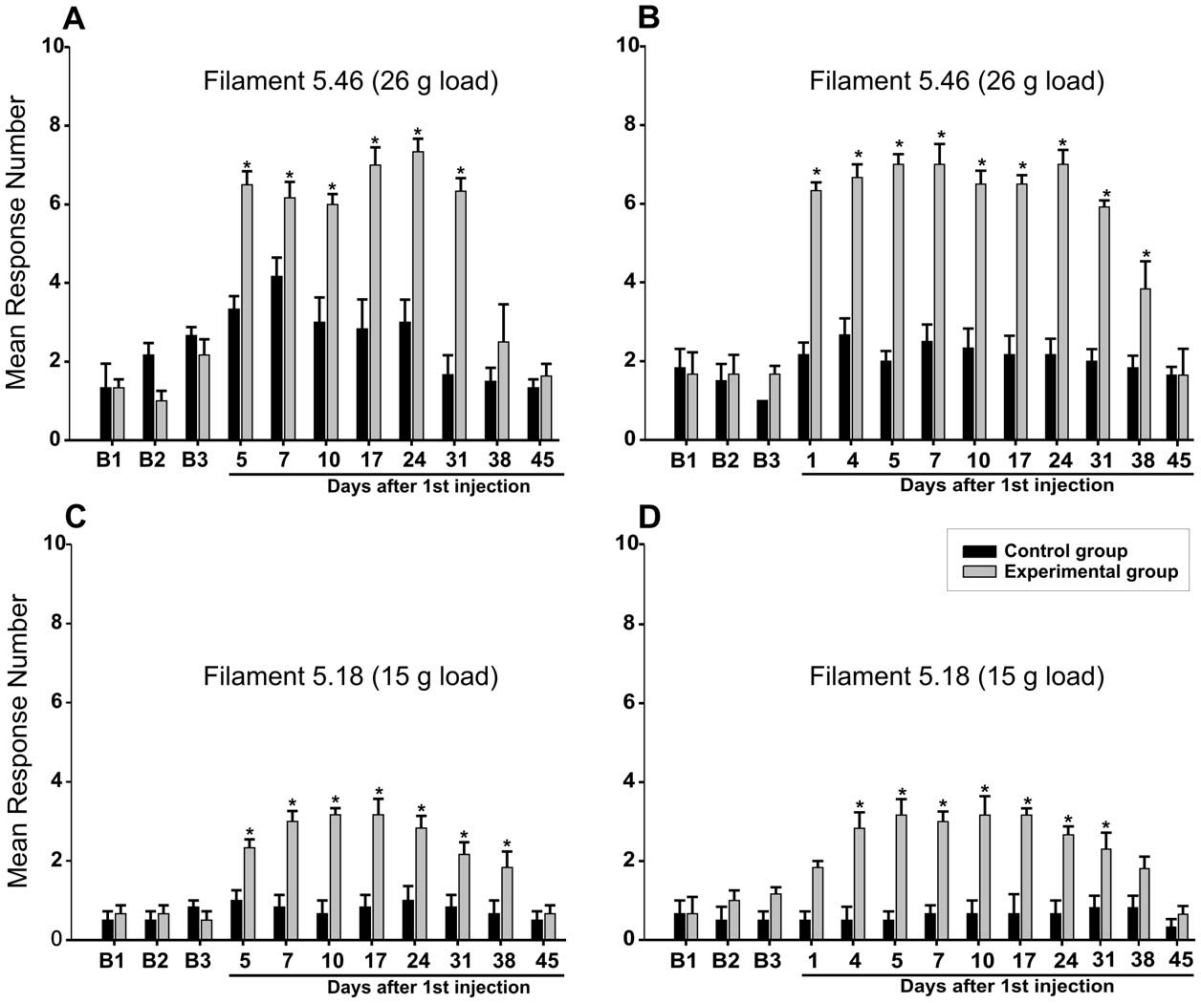
Acidic saline injections increase nocifensive behaviour. Animals received two injections of either normal saline (Control group, black bars) or acidic saline (Experimental group, gray bars) into both masseter muscles. The injections were made 2 days apart. Pressure was applied to the centre of the masseter muscle with von Frey filaments, and flinching and/or head withdrawal was scored as a positive response. Each muscle was tested 10 times with each of the filaments, and data from left and right were averaged. Means and standard errors are shown for three days prior to injection (B1, B2 and B3), and for 45 days after the first of the two injections. A, C: postinjection testing began 2 days after the second injection (n = 6). B, D: testing of these animals (n = 6) began 1 day after the first injection. *, p<0.05.

Figure 1 shows data gathered from two batches of 6 male rats that were tested with von Frey filaments during the baseline period (B1–B3), and after two bilateral injections of normal (pH 7.2, Control group n = 6) and acidic (pH 4.0, Experimental group n = 6) saline into the masseter muscles on Days 1 and 3. Testing began in one batch of animals 5 days after the first injection (Fig. 1A, 1C); in the other, testing was done between the two injections. During the baseline period, filament 5.46 (26 g load) caused between 0 and 3 responses per side (Fig. 1A and 1B), while filament 5.18 (15 g load) caused even less (Fig. 1C and 1D).

Two-way ANOVA analyses were conducted separately for each batch. They showed that the effects of both time and treatment were significant (ps<0.0001), as was the interaction between them. The frequency of responses did not increase significantly in the Control animals after injection of normal saline. In contrast, responses to both filaments increased greatly in the Experimental group. However, as shown in Panel 1B and D the responsiveness to the stiffer filament (5.46) was significantly increased on the first day after the injection while it appeared after four days when the more flexible filament (5.18) was used. Simple contrast analyses showed that the differences between the Control and Experimental groups remained significant for between 31 and 38 days.

### Acidic saline injections activate early genes in NVmes neurons innervating muscle spindle primary afferents

**Figure 2:**
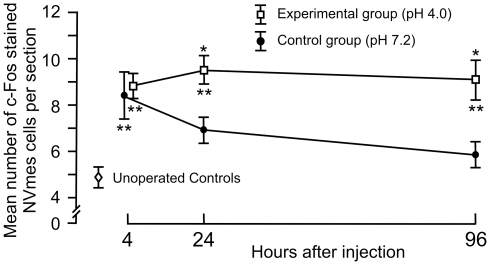
Normal and acidic saline injections increase the number of c-Fos expressing cells in NVmes, but only with acidic saline does the level of expression remain elevated. Graph showing the mean number ± S.E. of c-Fos stained NVmes cells in Unoperated Controls (open diamonds), and Experimental (open squares) and Control group (filled circles) at 4, 24 and 96 hours after injection. Asterisks above points: significance compared to the Control group. Asterisks below points: significance compared to Unoperated Controls. *, p<0.05; **, p<0.01.

In order to see if masseter muscle spindle Ia afferents could be involved in the development of muscle tenderness after intramuscular acidic saline injections we decided to use the expression of the early gene c-Fos in their cell bodies as a molecular marker of neuronal activation. Two groups of rats were given single bilateral injections of acidic (Experimental group, n = 14) or normal saline (Control group n = 14). The number of neurons immunoreactive to c-Fos within NVmes, which holds the cell bodies of the spindle afferents, was counted in animals sacrificed 4, 24 and 96 hours after the injection, and also in 5 Unoperated control rats. NVmes primary afferent neurons are easily distinguished from surrounding neurons in the brainstem because of their large, round or oval cell bodies and lack of dendrites. In the Unoperated Controls, the mean number of c-Fos labelled NVmes neurons per section was 4.91±0.5. The count was significantly higher 4 h after intramuscular injections in both the normal saline Control and acidic saline Experimental groups (Tukey's tests; ps<0.01; Fig. 2). However, at 24 and 96 h post-injection, the cell counts of the normal saline Control group did not differ any longer from those of the Unoperated Controls, whereas those of the Experimental group remained significantly higher (Tukey's tests; ps<0.0001; Fig. 2). Two-sample t-test comparisons showed that the counts in the Experimental group were also significantly higher than those of the normal saline Control group at 24 hrs (p<0.03) and 96 hrs (p<0.05).

### Acidic saline injections cause long-term changes in membrane properties of primary muscle spindle afferents

To test whether the increased level of activity suggested by the c-Fos experiments resulted from changes in NVmes cell basic membrane properties, we performed whole cell patch recordings in three groups of animals that all received two injections of saline at two day intervals, as in the behavioural experiments. Group A was given bilateral injections of normal saline. In Group B, one masseter received acid, the other normal saline, while Group C received bilateral acidic saline (Table 1). Animals were sacrificed at various times (<1–35 days) following the second injection. Brainstem slices were prepared and whole cell patch clamp recordings from NVmes cellbodies were carried out in order to measure a number of variables associated with the action potential (spike amplitude and half width; after hyperpolarization amplitude and duration) and membrane properties [resting membrane potential (RMP), input resistance, amplitude of high frequency oscillations] and thresholds for inward and outward rectification, high frequency oscillations, firing and bursting.

**Table 1:** Long-term effects on NVmes neurons: Animal groups and neuronal populations.

Animal group	A (n = 17)	B (n = 37)		C (n = 18)
Treatment	Normal saline	Normal saline	Acidic saline	Acidic saline
Side/Neuronal population	Bilateral/1	Unilateral/2	Unilateral/3	Bilateral/4
Size of neuronal population	54	87	100	67

Cells recorded from animals from each of the groups were divided into four populations, depending on treatment of the muscle that they innervated. The numbers of animals in each group and cells in each population are given.

A total of 308 NVmes neurons were used in the analyses. We first looked for differences in the 12 measured variables between initial neuronal populations that innervated muscles exposed to normal saline (neuronal populations 1 and 2 in Table 1) by using two-way ANOVA for Population and Time, then we did the same with the Experimental populations (neuronal populations 3 and 4). Population alone had no significant effect in any of the comparisons made for the normal saline neurons. There was a significant (p = 0.0065) difference in spike threshold between Population 3 (unilateral acidic saline) and 4 (bilateral acidic saline). However, the effect was small (mean ± SE = −53.2±5.5 for population 3 vs. −54.9±4.5 for population 4), and as it was the only significant effect out of the 12 comparisons, we decided to form one Control and one Experimental group to simplify further analysis.

**Figure 3:**
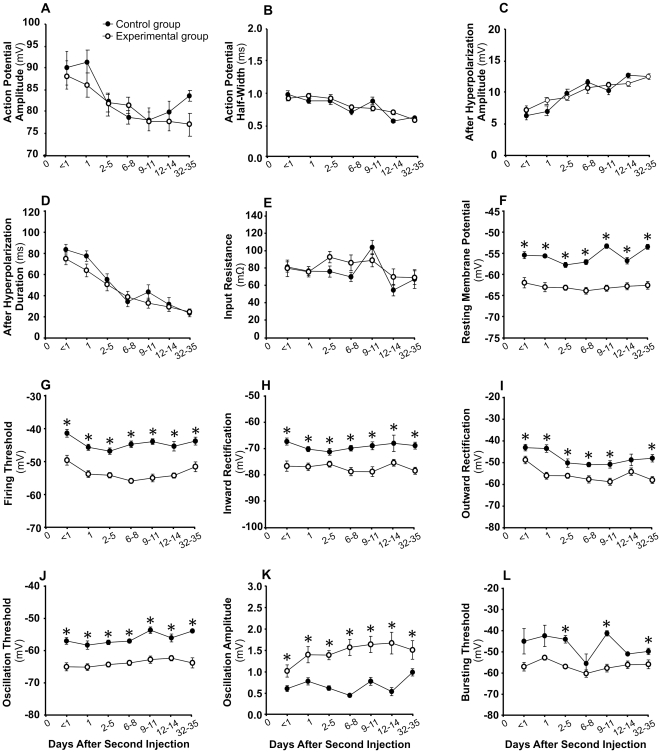
Acidic saline injections produce long lasting changes in many of the electrophysiological properties of NVmes cells innervating masseter muscle spindles. Means ± S.E. of twelve electrical properties are shown for Control (filled circle) and Experimental (open circle) neurons recorded at 7 time periods after the second intramuscular injection. *, p<0.05 for simple contrasts. In panels F, G and J, all ps<0.01.

Figure 3 shows that the properties of the action potential and after-hyperpolarization changed significantly over time (Fig. 3A–D). There was also a small but significant effect of time on mean input resistance (Fig. 3E), but group treatment had no effect on these 5 parameters (Table 2). Apparently, regarding these parameters, developmental changes in neuronal properties did not seem to interfere with treatment effects. On the other hand, treatment did have a significant effect on 7 parameters (Table 2): RMP (Fig. 3F), firing threshold (Fig. 3G), threshold of inward (Fig. 3H) and outward (Fig. 3I) rectifications, oscillation threshold (Fig. 3J) and bursting threshold (Fig. 3L) were all shifted in a more hyperpolarized direction, and oscillation amplitude increased (Fig. 3K). The differences between the two groups were clearly present within the first 8 h after the second injection (days<1), and they were generally maintained throughout the experiment (Fig. 3F–3L). The effect of time on these 7 parameters was relatively small, but was significant for all (Table 2) except inward rectification threshold (Fig. 3H) and oscillation amplitude (Fig. 3K). There was only one significant interaction (RMP) between group and time (Table 2).

**Table 2:** Long term effects of acidic saline on electrical properties of NVmes neurons.

VARIABLES/GROUPS	Control (n = 141)	Experimental (n = 167)	Group	Time	Group*Time
Action Potential Amplitude, mV	83.05±1.02	81.50±0.97	0.18	<0.0001	0.44
Action Potential Half-Width, ms	0.79±0.02	0.81±0.02	0.47	<0.0001	0.16
After Hyper-Polarization Amplitude, mV	10.32±0.28	10.34±0.22	0.93	<0.0001	0.11
After Hyper-Polarization Duration, ms	47.85±2.63	43.75±2.38	0.11	<0.0001	0.61
Input Resistance, MΩ	77.28±2.90	81.62±3.12	0.29	0.002	0.31
Resting Membrane Potential, mV	−55.80±0.27	−63.30±0.31	<0.0001	0.002	0.034
Firing Threshold, mV	−44.64±0.42	−53.89±0.40	<0.0001	<0.0001	0.30
Inward Rectification Threshold, mV	−69.51±0.55	−77.27±0.57	<0.0001	0.63	0.47
Outward Rectification Threshold, mV	−48.15±0.68	−56.09±0.62	<0.0001	<0.0001	0.28
Oscillation Threshold, mV	−56.25±0.35	−63.86±0.38	<0.0001	0.003	0.43
Maximum Amplitude of Oscillation, mV	0.66±0.03	1.49±0.07	<0.0001	0.19	0.14
Bursting Threshold, mV	−46.48±1.20	−57.25±0.69	<0.0001	0.013	0.13

Columns 2 and 3: mean ± SE of measurements made for each variable for the two groups. Columns 4–6: probabilities of alpha-type errors associated with the two-way ANOVA analyses are given for the effects of Group, Time and their interaction.

**Figure 4:**
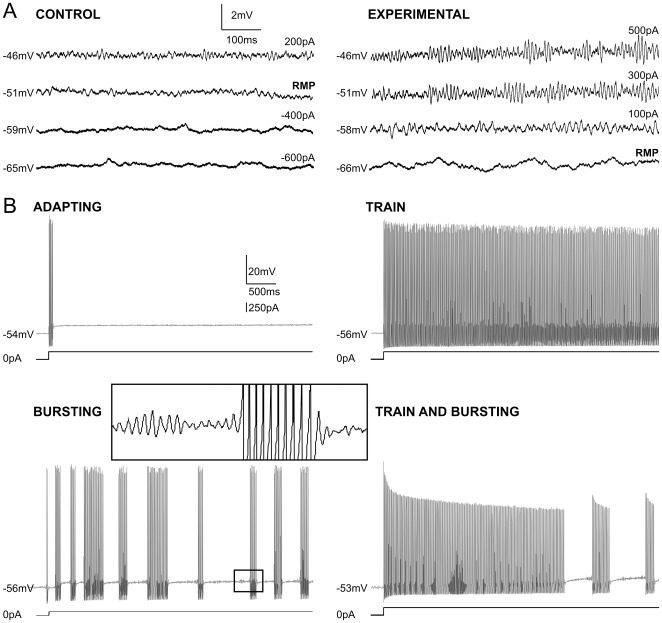
A: At equivalent membrane potentials, the subthreshold membrane oscillations of NVmes cells exposed to acidic saline are larger and the number of cells firing repetitively is greater. Examples of recordings made 14 days after the second injection in Control (left) and Experimental neurons showing the difference in oscillation amplitude at similar membrane potentials. The amount of current injected is given on the right of each trace. Calibration bars in the Control panel also apply to the Experimental panel. B: Examples of the four types of firing patterns induced by current injections. The inset illustrates a section of the “Bursting” trace at higher magnification to show that the bursts coincided with period of high amplitude oscillations. Calibration bars in top left panel apply to all panels in B. RMP: resting membrane potential.

Figure 4A shows examples of Control and Experimental neurons held at different membrane potentials to illustrate the lower threshold and greater amplitude of the rapid oscillations in the Experimental group.

### Acidic saline injections cause changes in firing patterns of muscle spindle primary afferents

An important finding was that 17 of the 167 NVmes cells (10%) from the Experimental group fired spontaneous action potentials at RMP, whereas none of the Control cells were active. This difference was highly significant (Χ^2^ = 16.9, df = 1, p<0.0001). We classified firing patterns induced by a 1 s suprathreshold depolarizing current injection into four types: Adapting, Train, Train and Bursting, and Bursting (Fig. 4B). Adapting neurons generated a single burst of spikes at the beginning of the step pulse that rapidly diminished in frequency, while Train neurons fired throughout the pulse. Bursting cells showed recurrent bursts of action potentials throughout the pulse, while a small number of neurons alternated between recurrent bursting and trains of spikes (Train and Bursting neurons). Bursting emerged from the high amplitude oscillations, as shown in the inset of Fig. 4B. The prevalence of these firing patterns in the Control and Experimental groups are given in Table 3. There was a small (15%) but significant between-group difference in the distribution of firing patterns (Χ^2^ = 9.99, df = 3, p = 0.019). In particular, a significantly greater percentage of Control neurons were of the Adapting type, and thus unable to sustain firing (Χ^2^ = 6.85, df = 1, p = 0.009).

**Table 3:** Long term effects of acidic saline on firing patterns of NVmes neurons.

Firing Pattern	Control (n = 141)	Experimental (n = 167)
Adapting (A)	91 (65%)	83 (50%)
Train (T)	29 (21%)	36 (22%)
Bursting (B)	17 (12%)	35 (21%)
Train/Burst (TB)	4 (3%)	13 (8%)
A	91 (65%)	83 (50%)
T+B+TB	50 (35%)	84 (50%)

There was a significant difference in distribution between groups when neurons were distributed into the four categories (Χ^2^ = 9.99, df = 3, p = 0.019), and also when grouped into Adapting and Non-adapting (T+B+TB) (Χ^2^ = 6.85, df = 1, p = 0.009).

We also compared the properties of NVmes cells in slices obtained from two Control (15 neurons) and two Experimental animals (18 neurons) that had completed behavioural testing, and that no longer showed signs of allodynia. The neurons were recorded 55 to 62 days after the second injection. Two-sample t-test analyses showed that none of the following parameters differed significantly between groups: RMP, firing threshold, input resistance, oscillation amplitude, and oscillation threshold. However, the inward and outward rectification thresholds were still significantly lower in the experimental group (p = 0.002 and p = 0.012, respectively). Bursting threshold could not be compared because only one neuron showed a bursting firing pattern.

### Single acidic injections modify membrane properties of muscle spindle primary afferents within 24 hours

The observations described above indicated that acidic saline injections were effective within 1 day following the 2^nd^ saline injection. Therefore, a second series of experiments was performed using only one muscle injection of either acidic (Experimental) or normal saline (Control). Two-way ANOVA showed that group assignment (Experimental, Control) had significant effects on RMP, firing threshold, thresholds for inward and outward rectification, oscillation threshold, and oscillation amplitude (Table 4). T-tests showed that all six variables were significantly lower than Control values 2 days after the injection, but only RMP and firing threshold were significantly lower on the 1^st^ day (Table 4).

**Table 4:** Short term effects of acidic saline on electrical properties of NVmes neurons.

	Control: 1-day Post-injection (n = 6)	Experimental: 1-day Post-injection (n = 8)	Control: 2 days Post-injection (n = 8)	Experimental: 2 days Post-injection (n = 9)	Group	Time	Group * Time
Action Potential Amplitude, mV	87.95±3.85	94.88±5.15 p = 0.29	88.02±2.35	79.51±3.62 p = 0.07	0.84	0.051	0.049
Action Potential Half-Width, ms	0.83±0.03	0.89±0.05 p = 0.3	0.81±0.03	0.93±0.05 p = 0.06	0.037	0.81	0.48
After Hyper-Polarization Amplitude, mV	9.16±1.46	5.59±1.13	9.28±0.73	8.77±0.99	0.080	0.15	0.19
After Hyper-Polarization Duration, ms	52.69±9.13	46.77±18.12	62.76±4.15	43.91±9.97	0.25	0.73	0.54
Input Resistance, MΩ	79.37±7.87	58.17±5.99	67.25±5.30	75.67±11.01	0.46	0.75	0.093
Resting Membrane Potential, mV	−53.88±0.97	−58.50±1.12 p = 0.01	−54.50±0.82	−58.22±0.78 p = 0.005	0.0001	0.85	0.63
Firing Threshold, mV	−40.56±2.85	−49.93±1.36 p = 0.01	−42.23±1.95	−53.91±1.05 p = 0.0003	<0.0001	0.17	0.56
Inward Rectification Threshold, mV	−64.13±2.42	−67.67±1.36 p = 0.23	−62.63±1.98	−73.11±3.14 p = 0.014	0.010	0.44	0.18
Outward Rectification Threshold, mV	−45.13±3.00	−54.00±3.29 p = 0.07	−41.12±3.92	−58.89±2.35 p = 0.02	0.0003	0.86	0.18
Oscillation Threshold, mV	−49.00±3.70	−55.33±1.48 p = 0.15	−53.63±1.24	−58.11±1.21 p = 0.02	0.023	0.11	0.68
Maximum Amplitude of Oscillation, mV	1.05±0.12	1.20±0.30 p = 0.66	0.76±0.08	2.01±0.17 p = 0.00004	0.0003	0.14	0.003
Bursting Threshold, mV	−30.25±5.86	−39.33±4.48	−41.00±5.86	−45.25±3.01	0.21	0.12	0.64

Mean ± SE of measurements made for each variable one and two days after a single bilateral injection of normal (Control) or acidic (Experimental) saline are shown. Probabilities of alpha-type errors associated with the two-way ANOVA analyses are given for the effects of Group, Time and their interaction. When the effect of Group or Group*Time were significant, t-tests were used for simple contrasts to compare Control and Experimental cells.

### Masseter muscle spindles contain small-calibre afferents that express nociceptor markers

**Figure 5:**
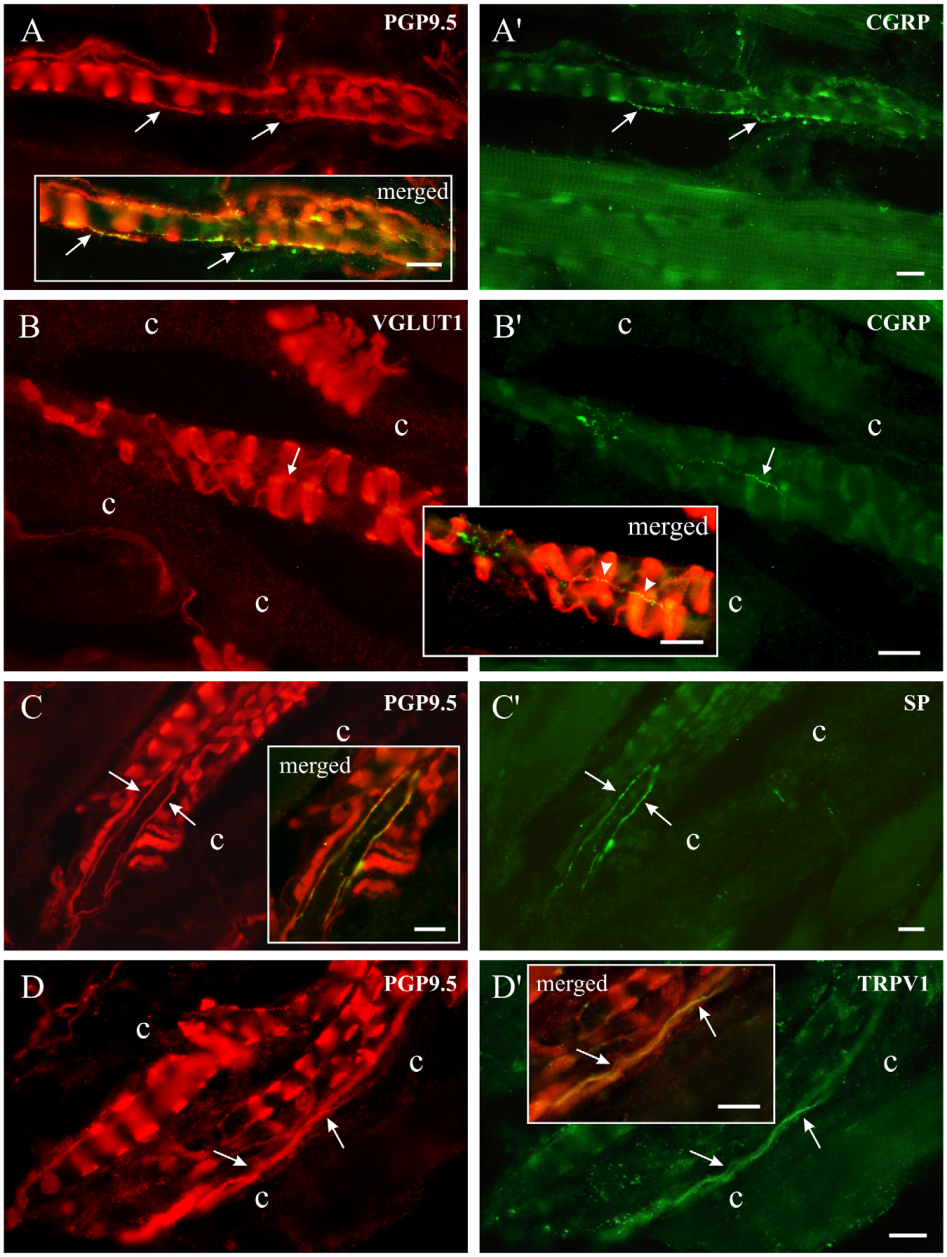
Masseter muscle spindles contain small-calibre afferents that express nociceptor markers. Left and right photomicrographs have identical frames with different sets of fluorescence filters. In each case, portions of the photomicrographs were digitally merged in boxed areas. A: Nerve fibres immunoreactive for PGP9.5 and containing CGRP (A'). B: A small green CGRP-positive fibre (B', thin arrow) runs across three VGLUT1-positive loops of an annulospiral ending. None of the VGLUT1-positive fibres in B corresponded to the CGRP positive fibres in B'. In the merged image, the fibre to the right appears yellow for most of its length (arrowheads) because it passes over red VGLUT1-positive fibres. C and C': Fibres immunoreactive for both PGP9.5 and SP. D and D': Fibres immunoreactive for PGP9.5 and the capsaicin receptor, TRPV1. c: spindle capsule wall. All scale bars = 10 µm.

One possible mechanism by which ectopic action potentials generated at the soma of NVmes neurons could lead to activation of nociceptive pathways would involve antidromic propagation of these action potentials back to the peripheral endings where they could cause glutamate release and activation of nearby nociceptive fibres. Physiological experiments designed to test this hypothesis would be challenging. Therefore, as an initial step, immunofluorescence was used to examine whether the machinery required to support this hypothesis is present. Thus, we looked for evidence that small-calibre fibres labelled with known nociceptor markers and glutamate receptors can be found in close proximity to muscle spindle annulospiral endings. Masseter muscles were removed from anaesthetized rats after perfusion with fixative, then sectioned and processed for immunofluorescence. Muscle spindles are very easy to identify in histological sections. The central equatorial region containing the annulospiral endings of the Ia primary afferents is particularly evident in longitudinal sections of spindles. These large-calibre endings and axons were all intensely labelled with PGP9.5 (Fig. 5A,C,D), a general marker of peripheral neuronal processes [28]–[31] and were also all labelled with VGLUT1 (Fig. 5B), a marker of glutamate containing fibres and terminals [32], [33]. It was already known that the annulospiral endings of Ia afferents in rat triceps surae [24] and masseter [23] contain VGLUT1.

**Figure 6:**
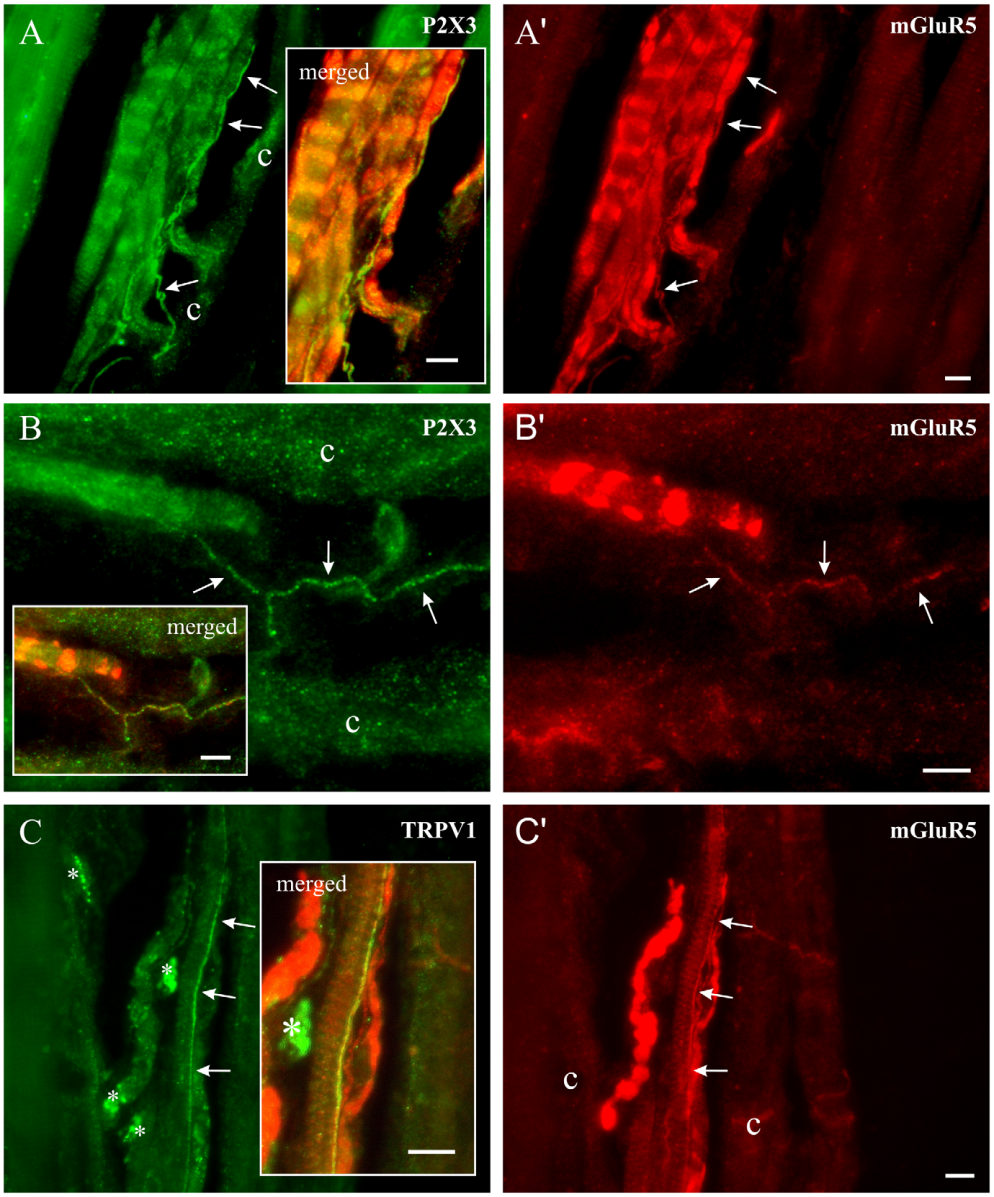
The putative nociceptors in muscle spindles carry mGluR5 receptors. In all cases, the photomicrographs from the left and right columns have the exact same frames but with different set of fluorescence filters. Merged portions of these photographs were placed in boxed areas to show the yellow-appearing double-labelled fibres. Left column shows photomicrographs of thin nerve fibres ( pointed by arrows) immunoreactive for P2X3 (A and B) or TRPV1(C). Right column shows immunoreactivity of these same fibers to mGluR5 (A', B', C'). *: fluorescent artefact. c: spindle capsule wall. All scale bars = 10 µm.

PGP9.5 also labelled small-calibre nerve fibres within spindles (Fig. 5A′,C′,D′), and most of these travelled parallel to the long axis of the intrafusal muscle fibres, in contrast to the large annulospiral endings, which encircle intrafusal fibres. Thin axons immunoreactive for PGP9.5 could be double labelled with several markers previously linked to nociceptors: CGRP (Fig. 5A′,B′), SP (Fig. 5C,C′), TRPV1 (Fig. 5D,D′) and P2X3, (Fig. 6A,B) [34]–[42]. These small-calibre fibres were clearly inside the spindles, often juxtaposed to intrafusal muscle fibres and annulospiral endings. They were not associated with blood vessels or the wall of the spindle capsule. CGRP or P2X3 fibres were the most often encountered in spindles, and intersecting in a number of places with the surrounding annulospiral endings. Some CGRP- and P2X3- immunoreactive fibres were continuously labelled, while others often appeared as a linear series of discontinuous fluorescent beads. Intrafusal small-calibre fibres immunoreactive for SP (Fig. 5 C′) or TRPV1 (Figs. 5D′, 6C) were sometimes seen. We found no examples of nerve fibres that bound the lectin IB4 within muscle spindles, although many small-calibre fibres were clearly labelled for IB4 in the masseter nerve. Spindle annulospiral endings were always negative for nociceptor markers.

**Figure 7:**
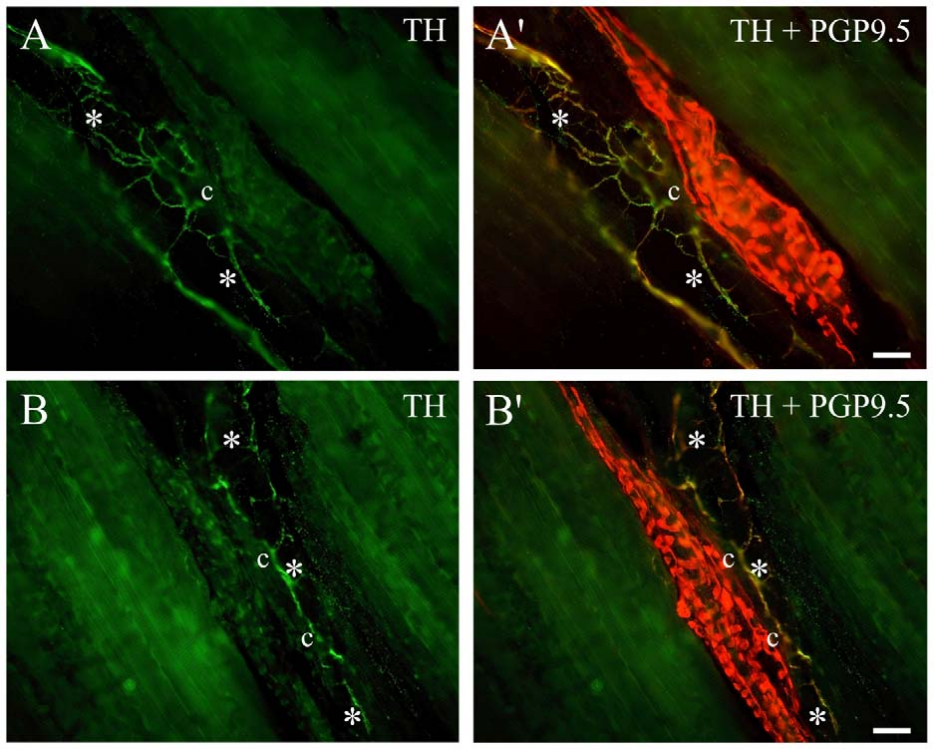
The thin axons in muscle spindles are not sympathetic fibers. Tyrosine hydroxylase (TH)-immunoreactive axons (light green in A, B; yellow in A',B') were seen close to muscle spindles and often over the capsule walls (c), but they were never seen among intrafusal muscle fibres. They were most often associated with blood vessels (*). Scale bars = 25 µm.

After finding small-calibre axons immunoreactive for nociceptor markers in close relationship with annulospiral endings, which are always positive for VGLUT1, we looked for the coexpression of nociceptor markers and glutamate receptor subunits mGluR5, NMDAR2B and GluR1. All annulospiral endings were intensely labelled for mGluR5, and many intrafusal small-calibre nerve fibres were also mGluR5-positive (Fig. 6A′–C′). The mGluR5 labelling appeared to completely fill some small axons with a fine granular reaction product; in others, it appeared as a string of fine beads (Fig. 6B′), which may indicate dispersed receptor units along the axons. Figure 6A shows a spindle with a clear annulospiral ending labelled for mGluR5 on the left, and two or more axons that co-express P2X3 and mGluR5 that run along the long axis of the intrafusal muscle fibres inside the capsule (c). Another double-labelled fibre shown in Fig. 6B eventually runs across the coils of an annulospiral ending labelled for mGluR5. The final example shows a mGluR5-positive TRPV1 fibre running along an intrafusal muscle fibre. We found no NMDAR2B or GluR1 immunoreactive products within spindles, but some well labelled NMDAR2B or GluR1 fibres were seen in the masseter nerve and around blood vessels (not shown). Although fine diameter axons within muscle spindles have generally been regarded as sympathetic fibres, e.g. [43], we found no tyrosine hydroxylase (TH)-containing fibres inside muscle spindles in any of the four muscles examined. TH-positive fibres were frequently found close to muscle spindles, but careful analysis showed that they were associated either with extrafusal blood vessels or with the equatorial spindle capsule wall (Fig. 7).

### Antagonists of glutamatergic receptors applied locally within the muscle prevent the acid saline-induced increase of nocifensive behaviour

**Figure 8:**
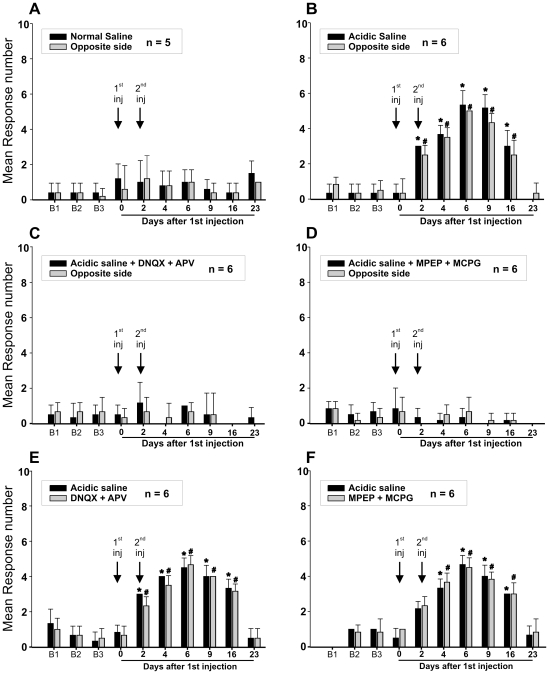
The increase in nocifensive behaviour induced by a unilateral injection of acid saline is bilateral and is prevented if antagonists of glutamatergic receptors are administered together with the acid solution, but not if they are injected into the contralateral muscle. Animals received two injections (arrows) of either normal (A) or acidic saline (B) into one of their masseter muscles. von Frey filament 5.18 (load = 15 g) was applied 10× on each side and the number of withdrawals was counted. Means and standard errors are shown. C: Ionotropic glutamate receptor antagonists (APV: 50mM and DNQX: 1mM) were added to the 2^nd^ injection. D: Metabotropic glutamate receptor antagonists MPEP and MCPG (1mM each) were added to the 2^nd^ injection. E: DNQX and APV were given contralaterally to the acid saline injection. F: MPEP and MCPG were given contralaterally to the acid saline injection. *, #: significantly different from their respective mean baseline (B1–B3) response.

If peripheral release of glutamate is involved in the allodynia observed in our animal model, then blockade of peripheral glutamate receptors should prevent or reduce it. To test this and to differentiate between peripheral and central mechanisms, we made unilateral masseter muscle injections of normal and acidic saline and measured nocifensive responses elicited by applications of von Frey filaments bilaterally. When compared to bilateral injections, unilateral injections of acidic saline (Fig. 8B) caused bilateral increases of nocifensive responses that were similar in magnitude, but that lasted for a shorter period, while normal saline had almost no effect (Fig. 8A). Mixtures of either ionotropic (DNQX and APV; Fig. 8C) or metabotropic (MPEP and MCPG; Fig. 8D) glutamate receptors antagonists given together with the unilateral injections of acid saline prevented the induction of allodynia on both sides of the face, but were ineffective if given contralaterally to the acid saline injection (Fig. 8E and F).

## Discussion

The results of our behavioural experiments suggest that bilateral as well as unilateral injections of acidic saline into rat masseter muscles induce a bilateral long-lasting allodynia-like response and cause increased immediate early gene c-Fos expression in the somata of the related muscle spindle Ia mechanoreceptor afferents. Within a similar time frame, the neurons developed changes in their electrical properties, which increased their excitability and caused a small but significant fraction to fire spontaneously *in vitro*. Furthermore, we confirmed that the Ia peripheral terminals contain high levels of the vesicular glutamate transporter VGLUT1 and discovered that small-caliber axons, which are located in close proximity to the annulospiral endings, express nociceptor markers and metabotropic glutamate receptors. Ipsilateral, but not contralateral, blockade of these receptors and/or of other peripheral glutamatergic receptors prevents development of the hypersensitivity.

### Validity of the model and clinical relevance

Injections of hypertonic saline have been extensively used to induce tonic pain in human muscles, including the masseters [44]–[46]. Several of these studies showed that descriptions of experienced pain quality, patterns of radiation and referral of pain, and associated motor and sensory symptoms were quite similar in experimental groups and in patient populations. However, hypertonic saline induced pain in humans dissipates rapidly once the infusion stops, and the changes in motor signs in animals reverse within 15 min [47]. In our study, we needed an animal model that allowed us to analyze the longer-term neural changes that may underlie the transition to persistent muscle pain and tenderness as seen in functional pain syndromes such as TMD, Myofacial pain and Fibromyalgia [8]. In humans, saline of pH 5.2 has been used to mimic the hyperalgesia and allodynia experienced following tissue acidosis and DOMS [12]. A model presented by Sluka et al. [13] fulfilled our requirements of expressing a long-lasting allodynia-like response (up to 30 days) in rats. Following two injections of acidic saline at pH (4.0), 2 to 5 days apart in the gastrocnemius muscle, they compared the effects of unilateral injections of saline at pH 4.0, 5.0, 6.0 and 7.2, and found that solutions at pH 4.0 produced a most reliable bilateral hyperalgesia. They also showed that intramuscular pH drops to 6.5 for about one minute after injection of 100 µl of saline at pH 4.0 and returns to control values within 7 minutes. In humans, exercise and inflammation cause a drop of pH that can be as low as 5 in muscles, and low pH is associated with pain [12], [48], [49].

Having adapted the methods of Sluka et al. [13] to the masseter muscles, we found that injections of saline at pH 4.0 cause a long lasting increase in mechanical sensitivity of these muscles as measured by the frequency of flinching and/or head withdrawals following application of von Frey filaments. The time course of the changes in mechanical sensitivity was similar to that reported by Sluka et al. [13] when using bilateral injections, but slightly shorter when using unilateral injections. In contrast to these results, Ambalavanar et al. [50] reported that repeated saline injections of pH as low as 3.0 had no effect on the pressure-pain threshold of rat masseters. This is puzzling since injections of solutions at pH 4.0 are painful in humans [12] and evoke allodynia-like reponses when injected into rat limb muscles [13], [14], [51]. Our lack of agreement with the results of Ambalavanar et al. [50] could be due to differences in the techniques employed. These authors measured withdrawal threshold with a rigid filament connected to a force transducer. Their figures suggest thresholds from 1 to 2 N, with great variability between animals and experiments. Like Sluka et al. [13], we compared the frequency of nocifensive responses to calibrated von Frey filaments, and found significant differences between Experimental and Control groups using forces equivalent to 0.15 and 0.26 N.

### Acidic saline muscle injections activate early genes in cell bodies of Ia muscle spindle afferents

As part of our investigation into the role of Ia muscle spindle afferents in development of long-term allodynia, we examined the level of expression the c-Fos protein in their cell bodies following muscle injection of acidic saline. c-Fos is a well established marker of neuronal activity that has been widely used to show neuronal groups activated by nociceptive inputs [52]. In particular, increases in c-Fos expression have been reported in studies of muscle nociception. Ro et al. [53] showed that a 10 min infusion of hypertonic saline into the masseter muscle of anaesthetized rats increases c-Fos expression in the medullary dorsal horn (caudal trigeminal nucleus) within 2 h. Taguchi et al. [54] reported a similar increase in the dorsal horn after eccentric contraction and compression of the extensor digitorum longus muscle. In our experiments the number of c-Fos-positive NVmes neurons increased within 4 h of injection both in Experimental and normal saline Control groups, but returned to the Unoperated Control level within 24 h in the normal saline Controls. This resembles the transient c-Fos expression in the spinal dorsal horn, which returns to base level 8–24 h after acute nociceptor stimulation [55]. However, c-Fos remained high for 96 h in the Experimental group, indicating that acidic saline causes an extended raise in expression of an early gene strongly linked to increased neuronal activity in DRG [56].

### Acidic saline muscle injections change Ia muscle spindle afferent membrane properties and patterns of firing

Experimental neurons had a lower RMP, and inward and outward rectification occurred at more hyperpolarized potentials. Furthermore, their high frequency membrane oscillations appeared at lower membrane potentials, the maximum amplitude of the oscillations was greater, and their threshold for firing was lower. Thus, neurons related to acid-exposed masseter muscles functioned at more negative membrane potentials, but yet were more excitable, and more likely to fire spontaneously than control cells. In most rodent neuropathic pain models, the increases in oscillation amplitude and spontaneous ectopic firing seen in the cell bodies of A-fibres, including muscle afferents were induced by direct injury to the axon [21], [57], [58]. We have found similar changes in muscle spindle afferents that may result from exposure of their terminals to H^+^, or to substances released from other cells in response to H^+^. Although the NVmes neurons have no ASIC3 channels or TRPV1 receptors [59], they do respond to pH drops *in vitro*. However, unlike trigeminal ganglion neurons they are insensitive to changes in pH between 7.4 and 6.0. Further reductions cause large current changes in NVmes neurons (but not in ganglion neurons), suggesting that they are more sensitive to low pH [41]. The NVmes neurons have distinctive electrical properties. Their strong inward and outward rectifications make it difficult for them to maintain firing during depolarization and also to maintain a hyperpolarized state [17]–[19], [60]. Strong outward rectification probably explains why most Control neurons adapted during maintained depolarization (Table 3). However, Experimental neurons discharged more during depolarization, and some of them fired spontaneously despite having a lower threshold for outward rectification and a more hyperpolarized RMP.

Spontaneous membrane oscillations, like those we have found in NVmes, were first described by Puil and Spiegelman [61] in trigeminal ganglion neurons. Later, Amir et al. [62] found that 35% of large DRG neurons showed oscillations between 88–195 Hz when depolarized. Unlike DRG and trigeminal ganglion cells, NVmes neurons receive synaptic inputs but their oscillations persist after blockage of their synapses [19]–[21]. All of these authors showed that the oscillations wax and wane in amplitude in an irregular manner (see also Figures 1 and 5). We confirmed that when NVmes cells are depolarized by current injection, firing occurs during the depolarizing crests of the oscillations [19]. Amir and Devor [63] found that non-oscillating DRG neurons, even when deeply depolarized, were unable to generate continuous discharge, signifying the importance of the oscillatory process for repetitive firing. Pedroarena et al. [20] reported seeing rapid oscillations in normal NVmes neurons only above −53 mV, which agrees well with our data on mean oscillation threshold of Control cells. However, the threshold of the oscillations was significantly lower in Experimental neurons.

The depolarizing phase of the oscillations in DRG and NVmes depends on a voltage-sensitive persistent inward sodium current (I_Nap_) [21], while the repolarizing phase requires an outward potassium current. Reports that wide spectrum blockers of voltage-dependent K^+^ channels, such as TEA and 4-AP, or intracellular infusions of Cs^+^, increase oscillation amplitude and repetitive firing in DRG neurons [64], suggest the involvement of K^+^ conductances in these processes. There is good evidence that ectopic firing in large-diameter spinal afferents is associated with long-term changes in the dorsal horn nociceptive pathways [6], [65], including a change in the composition of neurotransmitters that they release [66]. The substrate for similar changes exists in the brainstem because NVmes spindle afferents send collaterals into regions of the spinal trigeminal nucleus that also receive nociceptor inputs [67]. It has been shown that ectopic action potentials arising from central axons or somata of primary afferents can also travel antidromically towards the periphery [68]–[71]. Moreover, increased glutamate levels have been observed in the hind paw plantar skin following electrical stimulation of the sciatic nerve at intensities activating low threshold A-fibres. Thus, action potentials generated at the proximal part of the nerve cause release of glutamate within its peripheral skin innervation territory [72]. Although we have not yet demonstrated that this occurs in our experimental model, it is plausible that action potentials travelling antidromically from NVmes cell bodies into their Ia axons may induce a similar release of glutamate from their annulospiral endings. This would be similar to the “antidromic vasodilation” that result from stimulation of a dorsal root [73].

### Peripheral terminals of Ia muscle spindle afferents can release glutamate

Injections of glutamate and its agonists into muscles, including the masseter cause pain in humans [74], and excite nociceptive afferent fibres in animals [75]. Furthermore, blocking of glutamate receptors decreases nocifensive behaviors in animal models [53], and glutamate concentrations increase in human suffering from trapezius myalgia [76] and DOMS [77]. We suggest that some of this glutamate could come from muscle spindle mechanoreceptor terminals. Work on spindle afferents from limb muscles has shown that annulospiral endings contain synaptic-like vesicles [78] as well as synaptic vesicle proteins [79] and that they express the glutamate transporter VGLUT1 [24]. Furthermore Bewick et al. [80] showed that the endings release and recycle glutamate, and that the glutamate modulates their mechanical sensitivity. Therefore, it is probable that glutamate release from spindle afferent peripheral terminals also occurs in the masseter muscle because we and others [23] have shown that they also express VGLUT1. Alternately, glutamate can also be released from the nociceptors themselves [81].

### Masseter muscle spindles contain small-calibre nerve fibers expressing nociceptor markers

As mentioned in the Introduction, thin axons have already been described within muscle spindles and other mechanoreceptors including tendon organs, Ruffini endings and Meissner corpuscles [25], [26]. Paré et al. [27] showed that thin axons in Meissner corpuscles express peptides (CGRP and SP) and the vanilloid receptor, TRPV1, suggesting that they are nociceptive fibers. We have now discovered that similar nerve fibres within masseter muscle spindles also express nociceptor markers. CGRP and SP are two peptides that have been associated with nociceptors, but they have also been found in other types of primary afferents [34], [40], [82], [83]. Many of our masseter intrafusal axons were immunoreactive for CGRP, but only a few contained SP, which correlates well with the finding that 22% of trigeminal ganglion masseter afferents contain CGRP, but only 5% contain SP [84]. The ATP membrane receptor, P2X3, is mainly found in small-sized DRG neurons and it may be exclusively expressed by nociceptors [39], [85]. However, in the trigeminal ganglion, some medium-sized neurons are also labelled [86], [87]. We found many P2X3-positive small-calibre fibres in masseter spindles, in agreement with a report that 22% of masseter afferents, with cell bodies in the trigeminal ganglion, express this marker [86]. Some TRPV1-positive fibres were also seen within the muscle spindle capsule, although many more were seen around blood vessels. This capsaicin activated receptor is also strongly associated with nociceptors, and injections of capsaicin into the masseter are painful in humans [88]. The lectin IB4 binds to DRG nociceptor neurons [38] and to many P2X3- positive trigeminal ganglion neurons [86], [89], but we found no IB4-labelled afferents within our population of muscle spindles. Although somewhat surprising, our results are supported by a report that only 5% of trigeminal ganglion masseter afferents bind IB4 [84].

We have also found that both large and small-calibre fibres within the masseter muscle spindles express the glutamate receptors mGluR5. It is known that NVmes neurons express mGluR5 receptors from birth [90], [91] and we have now shown that the annulospiral endings bind mGluR5 antibodies. Many small-calibre P2X3 fibres were mGluR5-positive, and a smaller number expressed both TRPV1 and mGluR5. mGluR5 receptors have been shown to play a role in various types of pain including inflammatory, neuropathic and post-operative pain, and hyperalgesia [92]–[95] and it has been shown that peripheral mGluR5 antagonists attenuate nociceptive behaviour in rats [96]. This is in agreement with our results, which show that blockade of these receptors ipsilaterally to the side that received the acid saline muscle injection prevents development of allodynia bilaterally. However, central mechanisms must be involved in the contralateral sensitization produced by unilateral injections. Thus, it is clear that not only central but also peripheral release of glutamate is involved in modulating nociceptive transmission [22]. Obviously, there are many sources for release of glutamate within muscles. The possible contribution to persistent muscle pain of glutamate release from Ia annulospiral endings acting on intrafusal nociceptors remains an interesting but still open question.

### Conclusions

The results of our studies on the experimental acid-saline model for development of persistent muscle pain suggest that changes in the phenotype of muscle spindle primary afferent neurons could be linked to the development of allodynia and hyperalgesia as seen in Myofascial pain, Fibromyalgia, and Temporomandibular disorders. These phenotypic changes may lead to activation of central nociceptive pathways explaining the contralateral sensitization, but are also associated to ipsilateral changes involving sensitization of nociceptors through activation of their glutamatergic receptors. In the masseter muscle, at least some of these nociceptors have terminals within muscle spindles. Our results suggest that sensitization of these nociceptors involves activation of glutamatergic receptors and indicate that glutamate could be released by the annulospiral endings themselves, but do not allow to rule out the possibility that glutamate can be released from other sources as well.

## Materials and Methods

### Animals

All experiments were carried out on young male Sprague-Dawley rats, which were maintained and handled according to the guidelines of the Canadian Council on Animal Care. All protocols were approved by the animal care committees of the Université de Montréal and Umeå University (c-Fos experiments). Intramuscular injections were carried out under Isoflurane (Halocarbon Products, River Edge, NJ) anaesthesia.

### Effects of acidic saline injections on nocifensive behaviour

Twelve rats 37 days old were used for testing of allodynia of the masseter muscles. Six were given an injection of normal saline (Control group; 20 µl, 0.9% NaCl, pH 7.2) into the centre of each masseter muscle; the other 6 received bilateral injections of acidic saline (Experimental group; 20 µl, 0.9% NaCl, pH 4.0). Following the protocol established by Sluka et al. [13], the injections were repeated two days later. Sensory testing was carried out by an operator who was blind to treatment using a modification of the method described by Ren [97]. The animals were handled with a leather glove until they were comfortable nestling within it. A series of von Frey filaments (#s 5.07, 5.18, 5.46 and 5.88, which produce maximum loads of 10, 15, 26 and 60 g, respectively; Stoelting, IL, USA) were then applied to the centre of the muscle in order of increasing load. A filament was applied until it bent, and was maintained in position until the animal flinched and/or turned the head, or for a maximum of 1 s. Testing with each filament was repeated 10 times per muscle with a 2 second interval between trials, and the number of responses was noted. In 3 Control and 3 Experimental rats, testing was done on each of three days prior to the injections, then on days 5, 7, 10, 17, 24, 31, 38 and 45 days after the first injection for all animals. In the other 6 animals (3 Experimental, 3 Control), additional testing was done on the day after each of the injections to look for early changes in responsiveness. Four animals from this study (2 Experimental, 2 Control) that had completed behavioural testing were also used for electrophysiological experiments.

In a second series of experiments, 35 animals were used to test the effects of unilateral injections and of the effects of glutamate receptors antagonists. One third of these animals served as Controls; 5 received a unilateral injection of 0.9% NaCl, pH: 7.2 and 6 received a unilateral injection of acidic saline (pH 4.0). One third received glutamate receptor antagonists dissolved in the acid saline solution unilaterally. In 6 of these a mixture of DNQX (1 mM) and APV (50 mM) was used, while in 6 others, a mixture of MPEP (5 mM) and MCPG (5 mM) was used. In the last third, injections of acid-saline were made on one side and injections of the antagonists (mixture of DNQX and APV, n = 6; mixture of MPEP and MCPG, n = 6) were made on the opposite side.

All injections were repeated 2 days after and testing with von Frey filaments was conducted as above.

### Effects of acidic saline injections on c-Fos expression

Thirty-three rats 13–16 days old were used for this part of the study. Five animals did not receive any injections and were used as Unoperated Controls. Fourteen were given injections of normal saline (Control group; 20 µl, 0.9% NaCl , pH 7.2) into each masseter muscle; the other 14 were given bilateral injections of acidic saline (Experimental group; 20 µl, 0.9% NaCl, pH 4.0). Each animal was deeply anaesthetized with Isoflurane and perfused intracardially with phosphate buffered saline (PBS: 0.1 M phosphate buffer containing 0.9% NaCl, pH 7.4) followed by 4% paraformaldehyde in PBS. Equal numbers of Control and Experimental animals were perfused 4 h (*n* = 8), 24 h (*n* = 10) and 96 h (*n* = 10) after the injections. The brainstem from superior colliculus to obex was removed and post-fixed in the same solution overnight and immersed in a solution of 0.1 M phosphate buffer, pH 7.4, containing 30% sucrose for at least 48 h at 4°C. Frozen transverse sections, 30 µm thick, were cut on a freezing microtome, collected and rinsed repeatedly in PBS, thereafter transferred to hydrogen-peroxide (0.5%) incubation for 30 min to eliminate endogenous peroxidase activity. Sections were then incubated for 48–72 h at 4°C in a 1∶2000 dilution of a polyclonal c-Fos antibody (c-Fos(4):sc-52, 23 Santa Cruz Biotechnology Inc., Santa Cruz, CA) in PBS containing 0.3% Triton X-100 and 5% normal goat serum. After three washes in PBS, sections were incubated for 1 h at room temperature in a secondary biotinylated anti-rabbit IgG (BA-1000, Vector Laboratories, CA, USA). Antibody detection was carried out using Vectastain ABC kit (Vector Laboratories) with 0.07% 3,3′-diaminobenzidine (Sigma, St Louis, USA) as the chromogen for 6–8 min to yield a brown reaction product. The sections were then mounted onto chrome-alum-coated glass slides, air-dried overnight, and cover-slipped with xylene-based mounting medium, and examined using bright-field microscopy with a Zeiss Axiovert 100 microscope (×20 objective lens). Neurons immunoreactive to c-Fos were recognized by the dark brown precipitate of the DAB reaction. As negative controls, some sections were processed as described above, but without the primary antibody. These sections were free of c-Fos staining, showing that labelling resulted from the primary antibody. An examiner who was blind to treatment counted the number of positively stained NVmes cells along the whole length of both left and right nuclei from the oculomotor nucleus to the rostral part of the trigeminal motor nucleus. Every second section was examined to avoid double counting of these large diameter neurons. Results were expressed as means ± SE per section.

### Effects of acidic saline injections on masseter muscle spindle afferent electrical properties

Prior to experiments, the somata of muscle spindle afferents in NVmes were retrogradely labelled by injecting Cholera toxin β subunit conjugated to Alexa Fluor 488 (Invitrogen, Carlsbad, CA) or DiI (Sigma) into both masseter muscles of all rats. Anaesthesia for all muscle injections was produced by hypothermia prior to day P5, and by inhalation of Isoflurane in older animals.

#### Effect of acidic saline injections on spindle afferent membrane properties: Long-term changes

Data was gathered from 72 rats. Following intramuscular injections of fluorescent Cholera toxin β subunit (see above) 1–3 days after birth, they were divided into three groups: Group A, bilateral injections of normal saline (10 µl, 0.9% NaCl at pH 7.2 ) into the masseter muscles; Group B injections of 10 µl of saline pH 7.2 into one masseter, and 10 µl of acidic saline (pH 4) on the opposite side (approximately equal numbers received the acidic saline on the left and on the right); Group C, bilateral 10 µl injections of acidic saline (Table 1). The first intramuscular injection of saline was given on the day following the Cholera toxin injection, the second two days later. Electrophysiological experiments were carried out at 7 different times after the second injection: T1: 2–8 hours (n = 7); T2: 1 day (n = 6); T3: 2–5 days (n = 14); T4: 6–8 days (n = 16); T5: 9–11 days (n = 9); T6; 12–14 days (n = 11); T 7: 32–35 days (n = 9). The person carrying out the recordings and data measurement was not blind to treatment, but did not know what changes in neuronal properties could be expected.

#### Effect of acidic saline injections on masseter muscle spindle membrane properties: Short-term changes

Because a preliminary analyses of data from the experiment described above showed significant differences between neutral and acidic saline neurons 2–8 h after the second saline injection, a second experiment was performed using only one injection. Six animals were prepared as for Groups A (bilateral normal saline) and C (bilateral acidic saline) above. Recordings were carried out one (T1) or two days (T2) after the injection. The person carrying out the recordings and data analysis was blind to treatment.

#### In vitro recordings

At the predetermined time, animals were decapitated, the brains were quickly removed and coronal brainstem slices (250–350 µm thick) were cut with a vibrating microtome (Leica VT100S) and placed in ice-cold, modified sucrose-based artificial cerebrospinal fluid (ACSF in mM; 225 sucrose, 3 KCl, 1.25 KH_2_PO_4_, 4MgSO_4_, 26 NaHCO_3_, and 25 D-glucose). They were transferred to a submerged-type chamber for whole cell patch-clamp recordings and continuously perfused with oxygenated (mixture of 95% O_2_ and 5% CO_2_) normal ACSF (in mM: 126 NaCl, 3 KCl, 1.25 KH_2_PO_4_, 1.3 MgSO_4_, 2.4 CaCl_2_, 26 NaHCO_3_, and 25 D-glucose) at room temperature. The slices were examined through a fixed stage microscope (Eclipse E600FN, Nikon) coupled with a 40× water immersion lens. Labelled cells were identified using epifluorescence and the recording electrode was positioned under infrared microscopy. Whole cell patch-clamp recordings in current-clamp mode were made using an AxoClamp-2B amplifier (Axon Instruments, Foster City, CA). Patch electrodes (3.5–8.5 MΩ) were pulled from borosilicate glass capillaries (1.00 mm ID, 1.12 mm OD; World Precision Instruments, Sarasota, FL) on a Sutter P-97 puller (Sutter Instruments Company, Novato, CA) and filled with a K^+^-gluconate-based solution (in mM: 140 K^+^-gluconate, 5 NaCl, 2 MgCl_2_, 10 HEPES, 0.5 EGTA, 2 ATP di(tris) salt hydrate, 0.4 GTP tris salt). All chemicals were supplied by Sigma.

**Figure 9:**
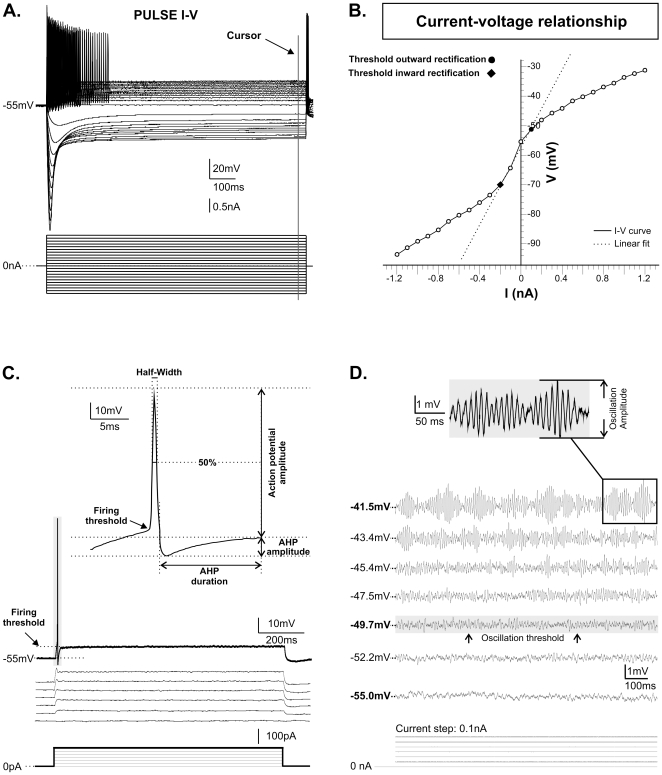
Methods used to describe membrane properties of NVmes neurons. A: Hyperpolarizing and depolarizing current injections of 1 s duration were used to construct current voltage relationships. A cursor was used to measure the trans-membrane voltage just before the end of the current step. B: The points of inflection on the I–V curve were taken as the thresholds for inward and outward rectification. C: Data from the depolarizing current pulses was also used to calculate firing threshold, action potential amplitude and half-width, afterhyperpolarization (AHP) amplitude and duration. D: Maximum oscillation amplitude (inset), and thresholds for high-frequency oscillations and for burst firing were also measured with the cursor.

Whole-cell current and voltage recordings were attained through a Digidata 1322A interface and analyzed with pClamp 9 software (Axon Instruments). Only cells with a resting membrane potential (RMP) below −50 mV were used in the experiments. The input resistance was measured from the slope of the linear portion of the I/V relationship (Fig. 9B) obtained by plotting membrane voltage changes produced by steps of current (0.1 nA, 1s; Fig. 9A). The membrane potentials at which there was an abrupt change in the slope of the I–V relationship (Fig. 9B,C) were taken as the thresholds of inward or outward rectification. In order to examine the basic firing characteristics of the cells, single spikes were evoked by brief intracellular pulses imposed (1 ms) on the holding potential. The following measurements were made: firing threshold, peak amplitude of the action potential from RMP, action potential half-width, amplitude of the after-hyperpolarization (AHP; measured from RMP), and AHP duration (measured as the time to RMP; Fig. 9C). Incrementing 1 s current steps were also used to measure the threshold of bursting when present and of the high-frequency membrane oscillations. Amplitude, oscillations (Fig. 9D) and frequency were measured at the membrane potential at which the amplitude appeared to be maximal. Means were calculated from three cycles with greatest amplitude (see inset). Patterns of spontaneous firing and firing during maintained depolarization were described.

### Anatomical relationship of muscle spindle mechanoreceptor and nociceptor afferents

Sixty-two rats 24 to 29 days old were deeply anaesthetized with pentobarbital (100 mg/kg IP) and perfused intracardially with 50 ml of PBS at 37°C followed by 200 ml of an ice-cold solution of 4% paraformaldehyde in PBS. The masseter muscles and trigeminal ganglia (used for positive controls of immunolabelling) were quickly removed, post-fixed for 90 min, and immersed in a 20% sucrose solution overnight at 4°C. The next day, the tissues were frozen in 2- methylbutane (Fisher Scientific, Nepean, ON, Canada) and kept at −80°C until processing. The muscles and ganglia were sectioned along their long axes at 15 µm, placed on ColorFrost Plus slides (Fisher Scientific), air-dried at 37°C overnight and processed for immunofluorescence the next day.

#### Immunofluorescence and lectin binding

All steps were carried out at room temperature unless specified otherwise. Tissue sections were rinsed three times for 10 min with PBS followed by a 60 min incubation in a blocking solution containing 6% normal goat serum and 0.3% Triton X-100 in PBS. This solution was also used for all antibody dilutions. Sections were then incubated overnight at 4°C in the presence of combinations of primary antibodies, or antibody and lectin. Rabbit anti-protein gene product 9.5 was used as a general marker of axons [28]. The choice of nociceptor markers and glutamate receptors was based on their reported presence in trigeminal ganglion neurons, particularly in masseter afferents, and on evidence that masseter inflammation can affect their expression [23], [24], [28], [84], [86], [98]–[102] (see also Table 5). The number of muscles used for each antibody and for combinations is given in Table 6.

**Table 5:** Sources of antibodies and lectin with final working dilutions.

Primary antibodies	Dilution	Company	Cat #
Rabbit anti-protein gene product 9.5 (PGP9.5); General marker of peripheral nerve fibres [26]	1∶500	Affinity Research	PG9500
Rabbit anti-vesicular glutamate transporter type 1 (VGLUT1); Present in annulospiral endings [22], [23]	1∶2000	Synaptic Systems	135 302
Sheep anti-calcitonin gene-related peptide (CGRP); Present in TG masseter afferents [81]	1∶500	Abcam	ab22560
Guinea pig anti-substance P (SP); Present in TG masseter afferents [81]	1∶1000	Chemicon	AB5892
Guinea pig anti-transient receptor potential vanilloid type 1 (TRPV1); Present in TG neurons [94]	1∶500	Neuromics	GP14100
Control peptide for TRPV1	20 µg/ml	Neuromics	P14100
Guinea pig anti-ATP-gated purinergic receptor subunit 3 (P2X3); Present in TG masseter afferents and affected by masseter inflammation [83]	1∶500	Neuromics	GP10108
Control peptide for P2X3	10^−5^ M	Neuromics	P10108
Rabbit anti-AMPA receptor subunit 1 (GluR1); Present in TG neurons and increased by masseter inflammation [95]	1∶2500	Immunostar Inc.	24439
Rabbit anti-NMDA receptor subunit R2B (NMDAR2B); Present in TG masseter afferents [96]; increased by masseter inflammation [95]	1∶100	Abcam	ab109
Rabbit anti-metabotropic glutamate receptor type 5 (mGluR5); Present in TG neurons; increased by masseter inflammation [95]	1∶500	Chemicon	AB5675
Control peptide for mGluR5	10 µg/ml	Chemicon	AG374
Chicken anti-tyrosine hydroxylase (TH); Marker of catecholaminergic fibres [97], [98]	1∶100	Chemicon	AB9702
**Lectin**			
Isolectin B4 from Griffonia simplicifolia conjugated to Alexa Fluor 488; Present in TG masseter afferents [81]	20 µg/ml	Invitrogen	I21411
**Secondary antibodies**			
Goat anti-rabbit-Alexa Fluor 594	1∶200	Invitrogen	A11012
Donkey anti-sheep-Alexa Fluor 488	1∶200	Invitrogen	A11015
Goat anti-guinea pig-Alexa Fluor 488	1∶200	Invitrogen	A11073
Donkey anti-chicken-FITC	1∶200	Jackson	703-095-155

**Table 6:** Number of muscles processed for each combination of antibodies and/or lectin.

Antibodies or lectin	PGP9.5	VGLUT1	CGRP	SP	P2X3	TRPV1	mGluR5	GluR1	NMDAR2B	TH	IB4
PGP9.5			9	3	4	6				3	4
VGLUT1			1	1	3	5				1	
CGRP	9	1					4	3	3		
SP	3	1									
P2X3	4	3					4				
TRPV1	6	5					3				
mGluR5			4		4	3					
GluR1			3								
NMDAR2B			3								
TH	3	1									
IB4	4										
Total muscles for each marker	29	11	20	4	11	14	11	3	3	4	4

**Figure 10:**
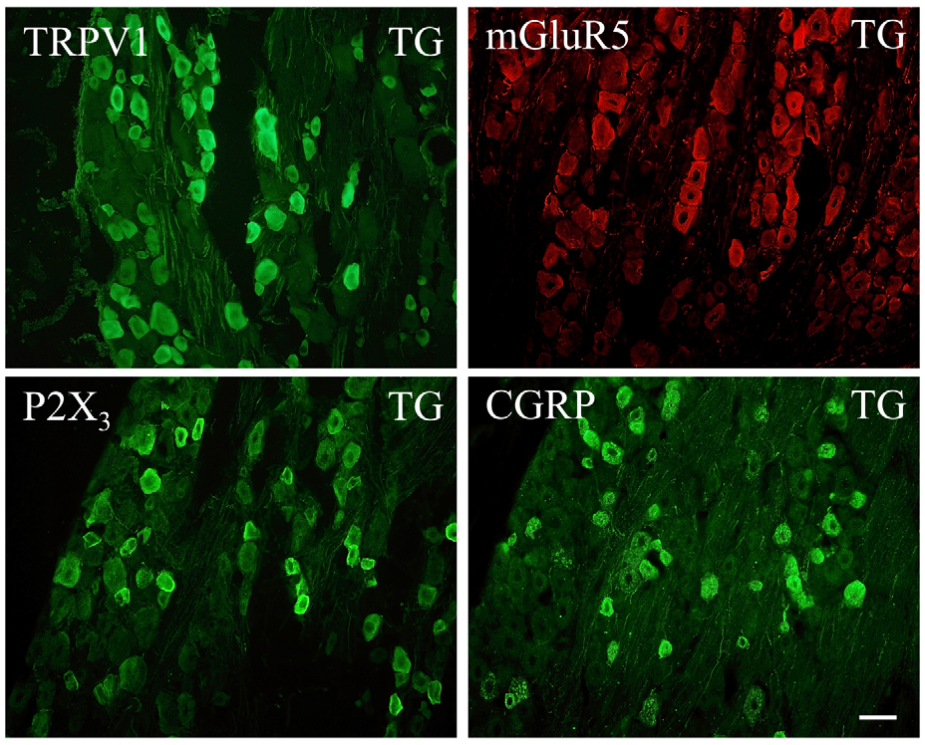
Labelling in the trigeminal ganglion was used as a positive control for antibodies tested in the masseter muscle. Photomicrographs of trigeminal ganglion neurons (TG) immunoreactive for four markers used in the present study. The scale bar (50 µm) applies to all photomicrographs.

The next day, the sections were rinsed three times for 10 min with PBS followed by incubation in the appropriate mixture of secondary antibodies for 60 min. All sections were then rinsed three times for 10 min with PBS. Those processed for immunofluorescence were quickly rinsed with distilled H_2_O and dried at 37°C for 15 min. Slides for lectin processing were incubated for 60 min at room temperature in a solution of distilled H_2_O containing MnCl_2_, MgCl_2_ and CaCl_2_, each at a concentration of 0.1 mM, and the IB4 lectin conjugated to Alexa Fluor 488 at 20 µg/ml. They were then rinsed with PBS followed by distilled H_2_O and dried at 37°C for 15 min. All slides were then cover slipped using Vectashield mounting medium (Vector Laboratories, Burlingame, CA, USA). All primary antibodies had been characterized by the manufacturer through pre-absorption tests, dot blots or Western blots (Table 5). Further control tests were carried out on trigeminal ganglia and on masseter muscles. In the negative controls, the primary antibodies were omitted, and in each case, there was no detectable labelling. Positive controls were carried out on trigeminal ganglia and results were compared to published studies to confirm that each antibody bound to the appropriate neural targets. Examples showing labelling of ganglion neurons with antibodies against TRPV1, P2X3, mGluR5 and CGRP are shown in Figure 10.

**Figure 11:**
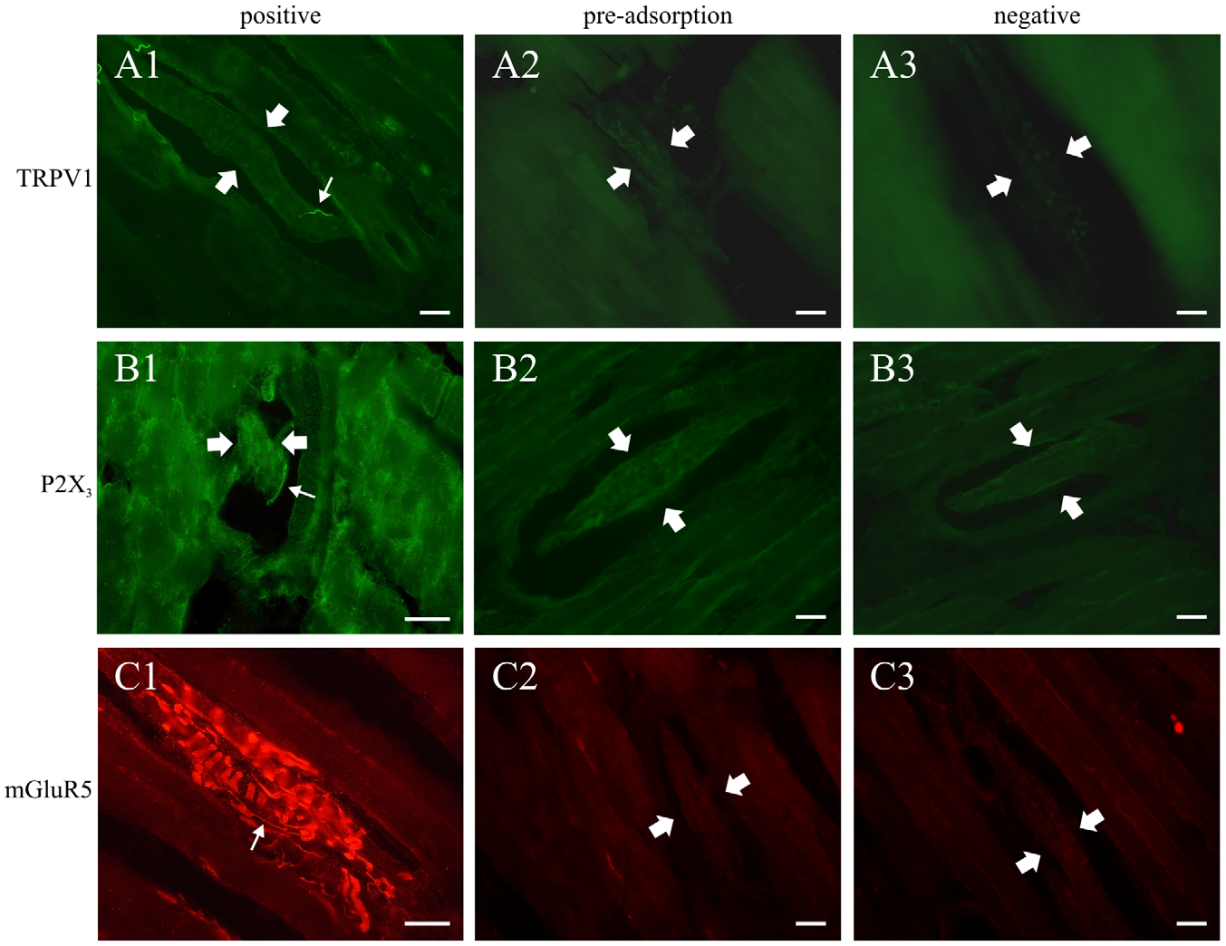
Preabsorption with immunogen peptide eliminated labelling in the ganglion and in the muscle indicating that the obtained labelling is specific. Photomicrographs of masseter muscle spindles showing positive, preabsorption and negative controls for three markers used in the study. Large arrows indicate the location of the spindle, small arrows point to small-calibre intrafusal nerve fibres. B1 and C1 were taken with a 60× objective, all others with a 40×. All scale bars = 25 µm.

As a further control, the TRPV1, P2X3 and mGluR5 antibodies were pre-absorbed using their corresponding immunogen peptide. Exposing the antibodies at final dilutions to 20 µg/ml, 10^−5^ M and 10 µg/ml of their respective immunogen peptide for 2 h at room temperature completely abolished their specific labelling of trigeminal ganglion neurons and their processes, including those in masseter muscle spindles (Fig. 11).

The sections were viewed under an Eclipse 600 epifluorescence microscope equipped with a DXM1200 digital camera (Nikon Canada, Montréal, QC). Adjustments of contrast and merges of photographs were performed using SimplePCI software (Compix, Cranberry Township, PA,USA).

### Statistical Analysis

Parametric data was expressed as mean ± Standard Error (SE). Normality of distribution was tested for all variables in all groups. When parametric tests could be used, between group comparisons were performed with ANOVAs or repeated measured ANOVAs. For pair wise comparisons, independent t-tests or Tukey's tests were used. Chi-squared tests were used to compare patterns of firing between treatment groups. Probabilities of alpha-type errors of <0.05 were considered to be significant. All analyses were carried out by a person naïve to the aims of the experiment.

### Limitations of the methods

For practical reasons, young animals are preferred for patch clamp recording, while older are better for behavioural experiments. However, even though most of the animals used for recording were injected with a tracer soon after birth, the effects of acid on membrane properties of NVmes neurons were still present when the animals were between 35 and 40 days old. This was at about the age of the animals used for the behavioural experiments, which began when animals were 37 days old. We suggest that the difference of a few days is unlikely to be important, particularly since the effect of time (and therefore age) on the key electrical parameters that were changed by acid exposure was weak or absent.
